# On the Use of Amalgan for Filling Teeth

**Published:** 1856-01

**Authors:** Elisha Townsend


### ON THE USE OF AMALGAM FOR FILLING TEETH.

BY ELISHA TOWNSEND.

It is somehow held to be more creditable to learn what one has not known, than to unlearn what he has erroneously believed. Blank ignorance shows a clearer account than posted mistakes, though it may not foot up any better at settlement day.

During his professional novitiate, the student is held innocent of that which he is faithfully pursuing, but has not yet attained; and the same gentleness of construction is allowed to cover the unknown, which lies beyond the acquisitions of the expert; but how embarrassing it is to a practitioner, with a dozen or a score of years of experience endorsing his diploma, to back square out of a mistake which he has been publicly pledged for!

Intending to make no excuse for my own tardiness in recanting a professional error, lest I should be betrayed into a justification of it, and inviting my brethren in the same category with myself to come up to the mark with as little reserve, I propose to give my present views upon the much vexed question of the propriety of the use of amalgams for filling teeth.

I have myself been so far ruled and over-ruled by authority in this matter, that I cannot honestly allow my own little influence to be responsible for its share, however small that may be, in the perpetuation of an injurious prejudice.

I cannot charge myself with either *great* rashness or *great* obstinacy in this matter, but on a full survey of all the points of principle and propriety, I can no longer withhold from the profession those clear conclusions which have now for some time governed my own practice, and must influence me until they shall be corrected by farther light and knowledge.

Without any more explanations I put myself into the witness box, and will be ready after the simplest delivery of my testimony *in chief;* for the *cross examination,* using both terms, however, in the sense which the lawyers give them, and neither allowing nor expecting any punning upon the words which imply punishment.

In 1834, I filled the posterior and buccal surface of an inferior dens sapientia for a professional friend with the amalgam of mercury and silver. It was considered impossible, from its position and frailty, to fill it with any thing else, and we supposed it might be retained for a year or two. It is now, though discolored, as good for a masticating organ as ever it was. The adjoining molar, which was apparently sound at that time, has since decayed and been removed. About the same time I filled a tooth for a clerical gentleman, which, though but a shell, he deemed very important. Of the success of this I gave him but little hope. I have seen it within three months, and it is as good as when filled. After a few more fillings, (all intended as experimental and to be carefully watched,) I abandoned the practice, not from a failure, so far as I was able to see in any of the operations I had performed, but because I was told that it was doing much harm, and that the good done by saving a few shells of teeth, even if they were saved, was more than counterbalanced by the injury inflicted upon the profession and the public by the quacks, who were authorized to use it by our example. This argument, used by men for whom in every way I had, and still have, the highest respect, and who stood in the foremost rank of the profession, made me willing to refrain from its use, and from that time until September, 1854, I never did use it.

My attention was then called to it by a professional friend in New York, who told me he had been making a series of experiments which had fully convinced him of its value, and also of its freedom from all the objections which had been urged against it by its opposers. These objections were—

1st. That it became black, and discolored the teeth in which it was placed. 2d. That it had produced salivation or ptyalism. 3d. That it contracted in hardening, and therefore did not fill the cavity, allowing moisture to surround it and reproduce decay.

The first of these objections did exist but is entirely removed by the present mode of preparing it, which consists, mainly, in adding a large portion of pure tin, and then washing the compound thoroughly with absolute alcohol. This, if carefully done, will insure its remaining almost as white as frosted silver.

The second objection, though answered with as much certainty, may not be so easy of conclusive proof. I have not been able to find any one, on whose judgment I could rely, and who really knew what ptyalism was, say he had met with a case of sure and marked character. Some had met with great tumefaction of the gums, looseness of the teeth, ulceration, &c., but we know that all these conditions are present in cases where no mercury has been employed. Stopping a carious tooth with a pledget of cotton, where there is disposition to alveolar abscess, will produce great swelling. Carelessness and want of cleanliness will allow accumulations of tartar and a consequent loosening of the teeth, accompanied by a fetor of the breath, equal in disagreeableness to the odor of ptyalism, and not very readily distinguishable from it in all cases.

I know of one case which was reported as one of decided salivation, and confirmed also by two physicians, which was said to come from four large amalgam fillings; the mouth was very filthy, and no care had been taken by the patient to cleanse it. She was told it was so much diseased that she must lose all her teeth, perhaps her life. This filthy mouth was cleaned, the gums properly treated, and entirely restored to health, without even removing the fillings on whose devoted heads the anathema had been poured. The mercury of the amalgam was, therefore, clearly not answerable for the symptoms in the case, and I have not been able to find any other that would better warrant the charge against the material, at least no case or fact which requires us to rule it out of practice on this apprehension.

3dly. It contracted in hardening, &c. This, by actual and careful experiment, it is proven not to do. It is well known that all substances or compounds which harden by the process of crystallization, rather expand than diminish their bulk. Now an amalgam of silver and mercury hardens by this process, and therefore cannot contract. That it does not contract is well proved, besides, by all experience of its use in dental cavities.

But, it will be urged, you endorse all the quacks in their empiricism by your example. Not at all; for it will be found to require as nice skill in the preparation of the cavity, as great care in the preparation of the material, and as much dexterity of manipulation in using it as are required in the employment of gold for the purpose. I would ask, does a physician, who gives calomel or arsenic judiciously, as he knows how to do from his teachings and experience, endorse the indiscriminate use of the same agents in the hands of the quack? is he in any way responsible? I think not.

Now, it is well known to all dentists of large practice, that cases are constantly coming into their hands where the cavity of decay is so situated as to be impossible to be certain that the particles of gold are placed in such apposition as effectually to exclude moisture and the chemical agents which produce caries, and in such cases, if what we claim for amalgam be true, it should take the place of gold; it can be packed closely and firmly, filling every part of the cavity without endangering the texture of the thinnest shell. If this can be done, as with this material we know it has been by very poor operators, how much more valuable does it become in the hands of the expert and careful manipulator? In a future paper I will give some cases in which I think it better than gold, with directions for the proper preparation of the material, and the mode of its employment and application. Now, I am only concerned to put in a plea for a material that I deem to have been unjustly put under ban by those who were unacquainted with any good there might be in it, and who, for the most part, saw it only in cases where, if the same operator had used gold or tin, the result would have been more disastrous to the teeth.

The following case is to the point: Twelve years since a lady came to me for professional advice and aid, and among the things she wished me to do, was to remove two amalgam fillings from her front incisors, put in three years before; the enamel was very thin, and slightly cracked; the fillings showed black through it. I told her I thought it would be impossible to get it out without breaking the teeth, as it was their principal support. These teeth I have watched, at intervals of six to eight months, ever since, until last spring, when the lady died of disease of the lungs. The teeth were serviceable to her to the last, and in appearance were as good as when I first saw them. I venture the assertion, that had they been filled with gold fifteen years since, even by the most expert operator, they would not, as they did, have served her through life.

I mention this as a case which clearly sustains all the points for which I offer it. It may not, perhaps, have been warrantable to fill a front tooth with a preparation which would become so black; but the object of filling teeth is to save them the longest time possible, with as little detriment to their appearance as possible; if this can be better done by a plastic material, than one which requires heavy pressure for consolidation, then the plastic material is the better; and if that material can be relieved of its objectionable feature of discoloring, we have another agent in our service, where it is believed to be preferable.

Neither do I fear, as some do, that it will render dentistry so easy that there will be laxity of moral feeling in the prosecution of our duty—for, if properly done, no time is saved to the dentist, no care lessened of manipulation in the proper preparation of the cavity, and a great deal of care and skill are necessary to pack and work the amalgam to a proper surface. I do not think, either, that it ever can or will supercede the use of gold foil, where gold can be used; but there are cases occurring in the practice of every dentist, in which, if he does his best duty to his patient, he is bound to use it, if he knows how.

Contenting myself, for the present, with the avowal rather than with the description, or ample examination of opinions stated, I wait another opportunity for presenting the results of experience and its teachings to my brethren in the profession, and I would be glad, in the mean time, to learn from them whatever they KNOW, for and against the practice.

P. S.—Since writing the above I have been made aware of the necessity of giving some directions now, as to the proper preparation of the amalgam for insertion, as I find some have been experimenting without being aware of the requirements to ensure success. Recipe—4 parts of pure silver; 5 parts pure tin. The silver to be melted in a crucible, and when partially cooled, the melted tin slowly added, carefully shaking the crucible while pouring in the tin; a black flux is then thrown in, and the whole is re-heated; then poured into the ingot. I am indebted for this method of preparation, to Dr. Wm. M. Hunter, of Cincinnati. It should be filed with a sharp keen file, which is kept for the purpose, and used for no other. A good magnet should then be carefully passed through it to remove any portions of steel that may have separated from the file. It is then to be bottled ready for use. After the tooth is perfectly prepared for filling, stuff it with cotton to exclude moisture, that you may more readily make the cavity perfectly dry when you have the composition ready, and lose no time, as it is needful it should be used as soon as possible after mixing. To mix it, take in the palm of the hand a globule of pure mercury, upon this put as much filings as you think will be sufficient to fill the cavity, rub these well together with the finger until they have thoroughly united, you then have a paste which is very soft and plastic, put it into a mortar, either glass or wedgewood, and put to it a teaspoonful of absolute alcohol; by triturating this you will soon find the alcohol become blackened, pour it off and add more, and so on, until the compound is thoroughly clean, then remove it from the mortar, dry it, and in a piece of chamois leather, or a twilled cloth, clear it of the superfluous mercury, by twisting in the fingers or squeezing in a vice. You then have a cake of white metal, which can be broken into pieces and be made to adhere to each other so as to form a uniform solid mass. In placing it in the cavity it should be used in small portions, taking care that the lower portions, or those in the bottom of the cavity, are firmly pressed down; in this way the cavity may be entirely filled, so as to leave it projecting a little beyond the surface. The burnisher may then be used to compress and smooth it, and alternately scraping the superfluous portions and burnishing until it has begun to harden, then it may be left until the next day, with direction to the patient not to use the side of the mouth until the next day; after twenty-four hours the filling may be filed and stoned and polished, as is usual with gold or tin fillings.

For packing the compound, I have found the point of a small file, such as is used for finishing plugs, the best instrument, and by softening the opposite end and thinning it, so as to make a sort of spatula of it, you have two instruments in one. I prefer to use it dry, and to keep the mouth and cavity dry while packing it as I do all kinds of filling; though I have been sometimes very successful even where the saliva reached it before finishing. Now I do not say, or think, that the method I have proposed, is the only, or it may be the best way, but for the present it is my method of using it, and I throw out these hints in the hope that the many energetic and scientific workers in our profession, will endeavor its improvement until it shall be all we desire. It is well known that several centuries since, the Chinese had a method of filling teeth with some material which was of the texture and color of enamel, but unfortunately the art is lost. Who knows but that some American dentist entering this field of the unknown, may recover this great secret, and thereby improve his art and bless mankind.—*Dental News Letter.*

